# Reformulated predictive torque and flux control with a full-order adaptive observer and accurate discrete-time models for sensorless induction machine drives

**DOI:** 10.1038/s41598-026-41944-y

**Published:** 2026-03-09

**Authors:** Ramón Herrera-Hernández, Carlos Reusser, Rodrigo Carvajal, Ramon Zamora

**Affiliations:** 1 School of Electrical Engineering, Pontificia Universidad Católica de Valparaíso (PUCV), Av. Brasil 2147, Valparaíso, 2362804 Valparaíso, Chile; 2 School of Engineering, Computer and Mathematical Sciences, Auckland University of Technology (AUT), 6 St Paul Street, Auckland City, 1010 New Zealand

**Keywords:** Induction machine, Model predictive control, Sensorless, Full-order adaptive observer, Discrete-time models, Engineering, Mathematics and computing

## Abstract

In this paper, we present a reformulation of both the predictive torque and flux control (PTC) scheme and the full-order adaptive observer (FAO) for induction machine drives. The proposed approach is based on a state-space representation expressed exclusively in terms of stator current and stator flux linkage, simplifying the observer structure and removing the explicit dependence on rotor flux variables found in conventional sensorless formulations. This representation is consistently applied within both the FAO and PTC frameworks, and second- and higher-order discrete-time models are derived using Taylor- and Runge–Kutta–based methods to enhance numerical accuracy and dynamic performance. The resulting FAO–PTC scheme is validated through Hardware-in-the-Loop simulations, demonstrating steady-state performance comparable to conventional designs, faster transient response, improved dynamic behaviour, and a reduced state-space order, albeit with slightly higher computational cost. Notably, simply employing a more accurate observer substantially enhances the performance of the sensorless scheme. Among the evaluated discretization strategies, the Taylor-based model provides the highest steady-state accuracy and fastest convergence, with only a modest increase in torque ripple. Overall, the proposed reformulated FAO–PTC framework achieves a balanced trade-off between accuracy, implementation simplicity, and computational efficiency for real-time sensorless induction machine drives.

## Introduction

Induction machines are the standard choice for demanding industrial applications due to their simplicity in construction, robust design, cost-effectiveness, and ability to operate across a wide speed range. Their efficiency and versatility have made them indispensable in various industries ^1^. To maximize their performance, advanced control strategies such as field-oriented control and direct torque control have become industrial standards. These methods enable high energy efficiency, recognized by their ability to achieve a high torque-per-ampere ratio and high performance at both steady and transient states ^1^. Among modern control methods, model predictive control (MPC) has garnered significant attention due to its fast dynamic response and straightforward implementation. Specifically, predictive torque and flux control (PTC), a control technique derived from finite control set-MPC and applied to drives, has demonstrated rapid dynamic behaviour and high-performance torque control ^2^. PTC employs a single cost function, where constraints are included straightforwardly, and the optimal voltage vector is selected based on the cost function, eliminating the need for pulse width modulation ^3^. Recent technological advancements have facilitated the implementation of MPC strategies in real-world applications, see e.g., ^3,4^. However, the accuracy of model-based control methods such as MPC heavily relies on having a precise dynamic discrete-time model of the controlled system. Small deviations in machine parameters, unmodelled dynamics, or inaccuracies in the model can degrade control performance ^5^.

In variable-speed drives, accurate knowledge of state variables such as currents, flux linkages, and angular speed is essential for achieving high-performance control. While these variables can be measured directly, doing so presents significant challenges. Speed sensors are highly susceptible to electromagnetic interference, require frequent maintenance, and are unsuitable for harsh environments ^6,7^. Similarly, electromagnetic flux sensors are difficult to integrate into machines and are prohibitively expensive. Consequently, speed and flux sensors are no longer commonly used. Instead, state variables are estimated using observers ^1^, relying solely on current measurements. Drives that operate in this manner are known as sensorless drives ^7^.

A key approach for achieving accurate sensorless control relies on model-based state estimation techniques, such as model reference adaptive systems ^8–10^, full-order adaptive observers (FAO) ^2,11–13^, Luenberger observers ^14,15^, sliding-mode observers ^16,17^, and Kalman-based filters ^18^, among others. Among these, the FAO is one of the most widely used approaches in induction machine drives. It relies on a linearized model of the induction machine to estimate stator current, flux linkage, and rotor shaft speed through the definition of an observer gain matrix and an adaptation law ^11^.

In conventional FAO formulations, rotor shaft speed estimation is typically implemented using a proportional–integral (PI) adaptive law driven by current or flux estimation errors. Recent studies have extended this classical approach by introducing iterative or numerical algorithms that build upon PI-based adaptation principles, aiming to enhance estimation accuracy and robustness under challenging operating conditions ^19,20^. These contributions primarily focus on the design of the adaptive mechanism, while preserving the underlying FAO structure and machine model.

Building upon these FAO-based sensorless schemes, several model predictive control (MPC) strategies employing FAO-based observers have been reported in the literature ^12,13^, where the emphasis is placed on observer modeling aspects such as core-loss representation and convergence properties. Similarly, ^21^ proposes a hybrid observer structure that combines adaptive and non-adaptive estimation schemes to extend the stability region of the observer.

In this context, accurate discrete-time models of the induction machine are fundamental to the effectiveness of model-based sensorless control strategies, as they enable precise system behaviour prediction in digital control implementations. The Euler method is widely used in machine drives for discretizing the continuous-time model and implementing sensorless control schemes due to its simplicity ^2,10,12,13^. However, higher-order methods such as Taylor and Runge–Kutta are less common because of their computational complexity. While Taylor discretization requires higher-order derivative calculations, Runge–Kutta approximates system evolution using intermediate points ^22^.

Despite their complexity, Runge–Kutta methods are extensively used for solving non-linear differential equations in simulations and are integral to software like Simulink and PLECS ^23,24^. Although the Euler method accurately represents machine dynamics at very high sampling rates ^25^, achieving such rates is often impractical due to hardware constraints. In contrast, higher-order Taylor and Runge–Kutta methods maintain accuracy even at lower sampling rates ^25^, making them a promising alternative for improving estimation and control performance without exceeding hardware limitations.

Motivated by these considerations, this paper investigates the use of high-order discrete-time models for sensorless predictive torque and flux control in induction machines employing a full-order adaptive observer. The proposed Taylor and Runge–Kutta models are designed to improve both control accuracy and estimation precision. Traditionally, machine drives are modelled using stator current and rotor flux linkage ^11–13^. In this work, we introduce a reformulated sensorless machine drive model that represents the system entirely in terms of stator current and stator flux linkage. This new formulation serves as the basis for developing discrete-time FAO and PTC models, ultimately reducing system complexity and minimizing the state vector.

## Induction machine continuous-time model

The dynamic equations that govern the time evolution of the induction machine (IM) in stationary reference frame $\alpha \beta$ are

1 $$\begin{aligned} \begin{aligned} \boldsymbol{{v}}_s^{{(\alpha \beta )}}&= R_s\,\boldsymbol{i}_s^{{(\alpha \beta )}} + \frac{d}{dt}\boldsymbol{\psi }_s^{{(\alpha \beta )}} ,\\ 0&= R_r\,\boldsymbol{i}_r^{{(\alpha \beta )}} + \frac{d}{dt}\boldsymbol{\psi }_r^{{(\alpha \beta )}} + p\,\omega _r\,\boldsymbol{J}\,\boldsymbol{\psi }_r^{{(\alpha \beta )}},\\ \boldsymbol{\psi }_s^{{(\alpha \beta )}}&= L_s\, \boldsymbol{i}_s^{{(\alpha \beta )}} + L_m\, \boldsymbol{i}_r^{{(\alpha \beta )}}, \\ \boldsymbol{\psi }_r^{{(\alpha \beta )}}&= L_r\, \boldsymbol{i}_r^{{(\alpha \beta )}} + L_m\, \boldsymbol{i}_s^{{(\alpha \beta )}},\\ H\,\frac{d}{dt}\omega _r&= \mathscr {T}_e - \mathscr {T}_l , \end{aligned} \end{aligned}$$

where $\boldsymbol{v}_s^{{(\alpha \beta )}}\in \mathbb {R}^2$ is the stator voltage in stationary $\alpha \beta$ reference frame; similarly, $\boldsymbol{\psi }_s^{{(\alpha \beta )}}$, $\boldsymbol{\psi }_r^{{(\alpha \beta )}}$, $\boldsymbol{i}_s^{{(\alpha \beta )}}$, $\boldsymbol{i}_r^{{(\alpha \beta )}} \in \mathbb {R}^2$ are the stator and rotor flux linkages and currents, respectively; $\omega _r \in \mathbb {R}$ is the rotor shaft speed; $\mathscr {T}_e$ and $\mathscr {T}_l$ are the developed electromechanical torque by the machine and the load torque at the rotor shaft, respectively. The stator and rotor resistances, $R_s$ and $R_r$ are assumed constant and balanced for $\alpha \beta$ components; likewise, the mutual inductance $L_m$ and the stator and rotor self-inductances, $L_s$ and $L_r$, are assumed constant and balanced. The equivalent inertia at the rotor shaft is *H*, and the total number of pole pairs is *p*. Further, $\boldsymbol{J}$ is a linear transformation

$$\begin{aligned} \boldsymbol{J}= \begin{bmatrix} 0 & -1 \\ 1 & 0 \end{bmatrix}. \end{aligned}$$

The developed electromechanical torque is expressed as

2 $$\begin{aligned} \mathscr {T}_e =\frac{3}{2}\,p \left( \boldsymbol{\psi }_s^{{(\alpha \beta )}}\times \boldsymbol{i}_s^{{(\alpha \beta )}} \right) , \end{aligned}$$

where $\times$ denotes the cross product of two vectors.

The currents and flux linkages are linearly dependent; therefore, it is possible to fully describe the IM dynamics using three state variables. Hereinafter, the state variables selected are the stator current, the stator flux linkage, and the rotor shaft speed, since the predictive torque and flux control (PTC) scheme predicts the stator currents and flux linkages to control torque. Hence, the IM state equation is

3 $$\begin{aligned} \begin{aligned} \frac{d}{dt} \boldsymbol{i}_s^{{(\alpha \beta )}}&= \left( a_{11} + a_{12}\,\omega _r\,\boldsymbol{J}\right) \,\boldsymbol{i}_s^{{(\alpha \beta )}} + \left( a_{21} + a_{22}\,\omega _r\,\boldsymbol{J}\right) \,\boldsymbol{\psi }_s^{{(\alpha \beta )}} + b_1\,\boldsymbol{v}_s^{{(\alpha \beta )}} ,\\ \frac{d}{dt} \boldsymbol{\psi }_s^{{(\alpha \beta )}}&= -R_s\,\boldsymbol{i}_s^{{(\alpha \beta )}} + \boldsymbol{v}_s^{{(\alpha \beta )}} ,\\ \frac{d}{dt}\omega _r&= \frac{1}{H}\left[ \frac{3}{2}p\,\left( \boldsymbol{\psi }_s^{{(\alpha \beta )}}\times \boldsymbol{i}_s^{{(\alpha \beta )}} \right) - \mathscr {T}_l\right] , \end{aligned} \end{aligned}$$

where the constants are

$$\begin{aligned} a_{11}&= -\frac{R_r\,L_s+R_s\,L_r}{\sigma L_s\,L_r},&a_{12}&= p ,\\ a_{22}&= \frac{R_r}{\sigma L_s\,L_r} ,&a_{22}&= -\frac{p}{\sigma L_s},\\ b_1&= \frac{1}{\sigma L_s} ,&\sigma&= 1-\frac{L_m^2}{L_s\,L_r} . \end{aligned}$$

Particularly, $\sigma$ is known as the total leakage factor.

Finally, in compact form, the dynamic IM model is described as

4 $$\begin{aligned} \frac{d}{dt} \boldsymbol{x} = \boldsymbol{f} (\boldsymbol{x},\boldsymbol{u}) , \end{aligned}$$

where (4) represent the IM model given by (3); $\boldsymbol{x}$ is the state vector and $\boldsymbol{u}$ is the input vector $\boldsymbol{v}_s^{(\alpha \beta )}$.

## Discretization methods

Given the discrete nature of modern controllers, it is essential to define a discrete-time model of the machine. In the literature, the Forward Euler method is the most commonly used discretization technique. However, it has been demonstrated that at low sampling times, the discrete-time model of IM derived using the Euler method exhibits significant inaccuracies ^25^. In this section, Euler, Taylor, and Runge-Kutta methods are reviewed.

### Euler discretization

The Euler method is intuitive and straightforward, and is the basis of the two other methods. It approximates the solution to the differential equation as follows

5 $$\begin{aligned} \boldsymbol{x}[k\!+\!1]= \boldsymbol{x}[k]+ T_s\frac{d}{dt}\boldsymbol{x}\bigg |_{\boldsymbol{x}[k]} , \end{aligned}$$

where *k* is the discrete time index, such that $\boldsymbol{x}[k]$ represents $\boldsymbol{x}$ at time $kT_s$, with $T_s$ a fixed sampling period.

### Taylor discretization

The derivative of a function is approximated using Taylor series expansions

6 $$\begin{aligned} \boldsymbol{x}[k\!+\!1]= \boldsymbol{x}[k]+ T_s\frac{d}{dt}\boldsymbol{x}\bigg |_{\boldsymbol{x}[k]} + \frac{T_s^2}{2}\,\frac{d^2}{dt^2}\boldsymbol{x}\bigg |_{\boldsymbol{x}[k]} + \frac{T_s^3}{3!}\frac{d^3}{dt^3}\boldsymbol{x}\bigg |_{\boldsymbol{x}[k]} + \cdots . \end{aligned}$$

Usually the series is truncated at the second derivative term, giving a second order approximation.

### Runge-Kutta discretization

The Runge-Kutta (RK) method approximates the solution of the differential equation based on estimates of the solution at different points. In this subsection two RK methods are taken into account: RK 2 and RK 4. The RK 2 approximation considers two estimates, whereas RK 4 considers four. The advantage that Runge-Kutta has over Taylor is that it is not necessary to explicitly calculate higher-order derivatives of the model.

#### Runge-Kutta 2

Runge-Kutta 2 is a second-order approximation. This method requires two evaluations of the system at each time step. For the IM

7 $$\begin{aligned} \boldsymbol{x}[k\!+\!1]= \boldsymbol{x}[k]+ \frac{T_s}{2} \left( \boldsymbol{r}_1+\boldsymbol{r}_2 \right) , \end{aligned}$$

and

8 $$\begin{aligned} \begin{aligned} \boldsymbol{r}_1&= \boldsymbol{f}(\boldsymbol{x}[k],\,\boldsymbol{u}[k]) , \\ \boldsymbol{r}_2&= \boldsymbol{f}\left( \boldsymbol{x}[k]+ T_s\,\boldsymbol{r}_1,\,\boldsymbol{u}[k\!+\!1]\right) , \end{aligned} \end{aligned}$$

where $\boldsymbol{u}[k\!+\!1]$ is the input vector at $kT_s+T_s$. Since the system is assumed to be causal, future inputs are unknown, therefore $\boldsymbol{u}[k]$ is used instead.

#### Runge-Kutta 4

Runge-Kutta 4 is a fourth-order method that requires four evaluations of the system in one sample period. The approximation of IM model by RK 4 is

9 $$\begin{aligned} \boldsymbol{x}[k\!+\!1]= \boldsymbol{x}[k]+ \frac{T_s}{6} \left( \boldsymbol{r}_1+2\,\boldsymbol{r}_2+2\,\boldsymbol{r}_3+\boldsymbol{r}_4 \right) , \end{aligned}$$

and

10 $$\begin{aligned} \begin{aligned} \boldsymbol{r}_1&= \boldsymbol{f}\left( \boldsymbol{x}[k],\,\boldsymbol{u}[k]\right) ,\\ \boldsymbol{r}_2&= \boldsymbol{f}\left( \boldsymbol{x}[k]+ \frac{T_s}{2}\,\boldsymbol{r}_1,\,{\boldsymbol{u}(t_k + \frac{T_s}{2})}\right) , \\ \boldsymbol{r}_3&= \boldsymbol{f}\left( \boldsymbol{x}[k]+ \frac{T_s}{2}\,\boldsymbol{r}_2,\,{\boldsymbol{u}(t_k + \frac{T_s}{2})}\right) , \\ \boldsymbol{r}_4&= \boldsymbol{f}\left( \boldsymbol{x}[k]+ T_s\,\boldsymbol{r}_3,\,\boldsymbol{u}[k\!+\!1]\right) , \end{aligned} \end{aligned}$$

where $\boldsymbol{u}(t_k + \tfrac{T_s}{2})$ represents the input value evaluated at the intermediate point between $t_k = kT_s$ and $t_k + T_s$.

Feeding the IM through a power converter implies that the input operates under a Zero-Order Hold (ZOH). Consequently, the intermediate points $\boldsymbol{r}_{2}$ and $\boldsymbol{r}_{3}$ use $\boldsymbol{u} [k]$ instead of $\boldsymbol{u}(t_k + \frac{T_s}{2})$ due to the ZOH assumption. Moreover, since the system is causal and future input values are unavailable, $\boldsymbol{u} [k]$ is also used in $\boldsymbol{r}_4$.

## Full-order adaptive observer

Since the drive is sensorless, only the stator current is measured; therefore, the remaining state variables are estimated using a state observer, specifically the full-order adaptive observer (FAO) ^11^. The proposed FAO employs a linear IM model represented solely by the stator current and the stator flux linkage as state variables, unlike the conventional FAO, which uses the stator current and the rotor flux linkage. The linear model of the IM used in the proposed FAO is given by

11 $$\begin{aligned} \begin{aligned} \frac{d}{dt} \underbrace{ \begin{bmatrix} \boldsymbol{i}_s^{{(\alpha \beta )}}\\ \boldsymbol{\psi }_s^{{(\alpha \beta )}} \end{bmatrix} }_{\textstyle \boldsymbol{x}}&= \underbrace{ \begin{bmatrix} a_{11}\boldsymbol{I} + a_{12}\omega _r\boldsymbol{J} & a_{21}\boldsymbol{I} + a_{22}\omega _r\boldsymbol{J} \\ -R_s\boldsymbol{I} & \boldsymbol{0} \end{bmatrix} }_{\textstyle \boldsymbol{A}} \begin{bmatrix} \boldsymbol{i}_s^{{(\alpha \beta )}}\\ \boldsymbol{\psi }_s^{{(\alpha \beta )}} \end{bmatrix} + \underbrace{ \begin{bmatrix} b_1\boldsymbol{I}\\ \boldsymbol{I} \end{bmatrix} }_{\textstyle \boldsymbol{I}} \boldsymbol{v}_s^{{(\alpha \beta )}} ,\\ \boldsymbol{y}&= \underbrace{\begin{bmatrix} \boldsymbol{I}&\boldsymbol{0} \end{bmatrix}}_{\textstyle \boldsymbol{C}}\,\boldsymbol{x} , \end{aligned} \end{aligned}$$

where $\boldsymbol{I}$ and $\boldsymbol{0}$ are the identity and null matrices of $2\times 2$, respectively.

The state observer is defined by

12 $$\frac{d}{{dt}}\hat{x} = \hat{A}\hat{x} + Bv_{s}^{{(\alpha \beta )}} + G\left( {\hat{i}_{s}^{{\alpha \beta }} - i_{s}^{{\alpha \beta }} } \right),$$

where the superscript $\,\hat{}\,$ denotes the estimation, and $\boldsymbol{G}$ is the observer gain matrix.

The FAO estimation error dynamic is

13 $$\begin{aligned} \frac{d}{dt}\boldsymbol{e} = \left(\boldsymbol{A} + \boldsymbol{G}\,\boldsymbol{C}\right) \boldsymbol{e} - \Delta \boldsymbol{A} \hat{\boldsymbol{x}} , \end{aligned}$$

where

$$\begin{aligned} \boldsymbol{e}&= \boldsymbol{x} - \hat{\boldsymbol{x}} ,\\ \Delta \boldsymbol{A}&= \hat{\boldsymbol{A}} - \boldsymbol{A}= \begin{bmatrix} a_{11}\,\Delta \omega _r\boldsymbol{J} & a_{22}\,\Delta \omega _r\boldsymbol{J}\\ \boldsymbol{0} & \boldsymbol{0} \end{bmatrix} ,\\ \Delta \omega _r&= \hat{\omega }_r -\omega _r . \end{aligned}$$

A Lyapunov function candidate is expressed as

14 $$\begin{aligned} V = \boldsymbol{e}^T\boldsymbol{e} + (\hat{\omega }_r-\omega _r)^2/\lambda , \end{aligned}$$

where $\lambda$ is a positive constant.

The time derivative of the Lyapunov function is

15 $$\begin{aligned} \frac{d}{dt}V = \boldsymbol{e}^T\left\{ \left( \boldsymbol{A} + \boldsymbol{G}\boldsymbol{C}\right) ^T + (\boldsymbol{A}+\textbf{GC})\right\} \boldsymbol{e} - 2\,a_{22}\,\Delta \omega _r \left( e_{i\alpha }\,\hat{\psi }_s^{(\beta )} - e_{i\beta }\,\hat{\psi }_s^{(\alpha )} \right) + 2\Delta \omega _r\frac{d}{dt}\hat{\omega }_r/\lambda , \end{aligned}$$

where $$e_{i\alpha } = i_s^{(\alpha )} - \hat{i}_s^{(\alpha )}$$, and $$e_{i\beta } = i_s^{(\beta )} - \hat{i}_s^{(\beta )}$$.

The adaptive speed scheme is designed such that the second and third terms of (15) cancel each other out, thus

16 $$\begin{aligned} \frac{d}{dt}\hat{\omega }_r = \lambda \, a_{22}\left( e_{i\alpha }\,\hat{\psi }_s^{(\beta )} - e_{i\beta }\,\hat{\psi }_s^{(\alpha )} \right) . \end{aligned}$$

Additionally, the observer gain matrix is selected so that the first right-hand term of (15) is non-positive, ensuring the convergence of the estimation. To improve the convergence of the adaptive speed scheme a proportional-integral (PI) is used. Therefore, the estimated speed is

17 $$\begin{aligned} \hat{\omega }_r = K_p\left( e_{i\alpha }\,{\hat{\psi }_s^{(\beta )}} - e_{i\beta }\,{\hat{\psi }_s^{(\alpha )}}\right) + K_i\int \left( e_{i\alpha }\,{\hat{\psi }_s^{(\beta )}} - e_{i\beta }\,{\hat{\psi }_s^{(\alpha )}}\right) \,dt , \end{aligned}$$

where $K_p$ and $K_i$ are the proportional and integral gains, respectively.

To select the observer gain matrix, the inherent stability of the IM model in (11) is exploited. The IM model is stable, with all its poles located in the left half-plane of the Laplace domain *s*. Based on this property, the observer poles are deliberately placed proportional to those of the IM model, ensuring that the observer preserves the stability characteristics of the machine dynamics.

The observer gain matrix is defined as

18 $$\begin{aligned} \boldsymbol{G} = \begin{bmatrix} g_1\boldsymbol{I} + g_2\boldsymbol{J} \\ g_3\boldsymbol{I} + g_4\boldsymbol{J} \\ \end{bmatrix} , \end{aligned}$$

and the observer poles are obtained by solving the characteristic equation

19 $$\begin{aligned} \det \left( s\boldsymbol{I} - \left( \hat{\boldsymbol{A}}+\textbf{GC}\right) \right) = 0 , \end{aligned}$$

where $\det (\cdot )$ denotes the determinant of a matrix.

To enforce proportional pole placement, the characteristic equation of the observer is matched to a scaled version of the characteristic polynomial of the IM model, yielding

20 $$\begin{aligned} s^4 -\eta \,2a_{11}\,s^3 + \eta ^2\,(a_{11}^2 + a_{12}^2\omega _r^2 + 2R_s a_{21})\,s^2 - \eta ^3\,\{ 2R_s(a_{12}a_{22}\omega _r^2 + a_{11}a_{21})\}\,s + \eta ^4\,\{ R_s^2(a_{21}^2 + a_{22}^2\omega _r^2)\} = 0, \end{aligned}$$

where $\eta$ is a proportional scaling factor. Since the poles of the IM are located in the left half-plane, choosing $\eta > 0$ guarantees that the observer poles remain stable.

By comparing the coefficients of the observer and IM characteristic polynomials, the matching procedure yields two distinct sets of solutions for the observer gain matrix, denoted as Solution I and Solution II in (21)

21 $$\begin{aligned} \begin{aligned}&\underline{\mathrm{Solution\,I}} & \underline{\mathrm{Solution\,II}} \\ g_1 =&(\eta - 1)\,a_{11} \;,&\qquad g_1 =&(\eta -1)\,a_{11}\,, \\ g_2 =&-(\eta +1)\,a_{12}\omega _r \;,&\qquad g_2 =&(\eta -1)\,a_{12}\omega _r \,,\\ g_3 =&R_s\left( 1-\eta ^2\,\frac{a_{21}^2 - a_{22}^2\omega _r^2}{a_{21}^2 + a_{22}^2\omega _r^2}\right) \;,&\qquad g_3 =&(1-\eta ^2)\,R_s \,,\\ g_4 =&\eta ^2\left( \frac{2\,R_s\,a_{21}\,a_{22}\,\omega _r}{a_{21}^2 + a_{22}^2\omega _r^2}\right) \;.&\qquad g_4 =&0 \,. \end{aligned} \end{aligned}$$

### Discrete-time models of the full-order adaptive observer

The FAO continuous-time model is given by the observer (12) and the adaptive scheme (17). The observer is discretized using the methods previously reviewed, whereas the PI is discretized using Euler in all cases.

#### Euler

Euler-based FAO is

22 $$\begin{aligned} \begin{aligned} \boldsymbol{i}_s^{{(\alpha \beta )}}[k\!+\!1]=&\left( T_s \left( a_{11} + a_{12}\omega _r\boldsymbol{J}\right) + 1 \right) \boldsymbol{i}_s^{{(\alpha \beta )}}[k]+ T_s( a_{21} + a_{22}\omega _r\boldsymbol{J} )\boldsymbol{\psi }_s^{{(\alpha \beta )}}[k]+ T_s b_1 \boldsymbol{v}_s^{{(\alpha \beta )}}[k],\\ \boldsymbol{\psi }_s^{{(\alpha \beta )}}[k\!+\!1]=&-T_s R_s \boldsymbol{i}_s^{{(\alpha \beta )}}[k]+ \boldsymbol{\psi }_s^{{(\alpha \beta )}}[k]+ T_s \boldsymbol{v}_s^{{(\alpha \beta )}}[k]. \end{aligned} \end{aligned}$$

#### Taylor

Second order Taylor-base FAO is

23 $$\begin{aligned} \begin{aligned} \boldsymbol{i}_s^{{(\alpha \beta )}}[k\!+\!1]=&\left\{ T_s\,c_1 - \dfrac{T_s^2}{2}\left[ R_s\,a_{21} - a_{11}\,c_1 - \omega _r(\,a_{12}c_1 - R_s a_{22})\boldsymbol{J} \right] + 1\right\} \boldsymbol{i}_s^{(\alpha \beta )}[k]+ \left\{ T_s c_2 + \dfrac{T_s^2}{2} c_1 c_2 \right\} \boldsymbol{\psi }_s^{ab}[k]\\ &+ \left\{ T_s b_1 + \dfrac{T_s^2}{2}\left[ a_{21} + a_{11} b_1 + \omega _r (a_{22} + a_{12} b_1 )\boldsymbol{J}\right] \right\} \boldsymbol{v}_s^{{(\alpha \beta )}}[k]+ b_1\dfrac{T_s^2}{2}\frac{d}{dt}\boldsymbol{v}_s^{{(\alpha \beta )}} , \\ \boldsymbol{\psi }_s^{{(\alpha \beta )}}[k\!+\!1]=&\left\{ -R_s\left( T_s + \dfrac{T_s^2}{2}c_1 \right) \right\} \boldsymbol{i}_s^{(\alpha \beta )}[k]+ \left\{ 1 - \dfrac{T_s^2}{2}R_s c_2\right\} \boldsymbol{\psi }_s^{(\alpha \beta )}[k]+ \left\{ T_s - \dfrac{T_s^2}{2}R_s b_1\right\} \boldsymbol{v}_s^{{(\alpha \beta )}}[k]+ \dfrac{T_s^2}{2}\dfrac{d}{dt}\boldsymbol{v}_s^{{(\alpha \beta )}}. \end{aligned} \end{aligned}$$

where $$c_1 = a_{11} + a_{12}\omega _r\,\boldsymbol{J}$$; $$c_2 = a_{21} + a_{22}\,\omega _r\,\boldsymbol{J}$$; and $$\dfrac{d}{dt}\boldsymbol{v}_s^{{(\alpha \beta )}} = \dfrac{\boldsymbol{v}_s^{{(\alpha \beta )}}[k] - \boldsymbol{v}_s^{{(\alpha \beta )}}[k\!-\!1]}{T_s}$$.

#### RK 2

Estimation points of RK 2-base FAO are

24 $$\begin{aligned} \begin{aligned} \boldsymbol{r}_1 =&\begin{bmatrix} \left( a_{11} + a_{12}\omega _r\boldsymbol{J}\right) \boldsymbol{i}_s^{{(\alpha \beta )}}[k]+ ( a_{21} + a_{22}\omega _r\boldsymbol{J} )\boldsymbol{\psi }_s^{{(\alpha \beta )}}[k]+ b_1 \boldsymbol{v}_s^{{(\alpha \beta )}}[k]\\ - R_s \boldsymbol{i}_s^{{(\alpha \beta )}}[k]+ \boldsymbol{v}_s^{{(\alpha \beta )}}[k]\end{bmatrix} , \\ \boldsymbol{r}_2 =&\begin{bmatrix} \left( a_{11} + a_{12}\omega _r\boldsymbol{J}\right) \left\{ \boldsymbol{i}_s^{{(\alpha \beta )}}[k]+ T_s\boldsymbol{r}_1(1)\right\} + ( a_{21} + a_{22}\omega _r\boldsymbol{J} )\left\{ \boldsymbol{\psi }_s^{{(\alpha \beta )}}[k]+ T_s\boldsymbol{r}_1(2)\right\} + b_1 \boldsymbol{v}_s^{{(\alpha \beta )}}[k]\\ - R_s \left\{ \boldsymbol{i}_s^{{(\alpha \beta )}}[k]+ T_s\boldsymbol{r}_1(1)\right\} + \boldsymbol{v}_s^{{(\alpha \beta )}}[k]\end{bmatrix} . \end{aligned} \end{aligned}$$

where $\boldsymbol{r}(i)$ is the *i*-th row of the vector $\boldsymbol{r}$

#### RK 4

RK 4 estimation point $\boldsymbol{r}_1$ is the same as RK 2, the others are

25 $$\begin{aligned} \begin{aligned} \boldsymbol{r}_2 =&\begin{bmatrix} \left( a_{11} + a_{12}\omega _r\boldsymbol{J}\right) \left\{ \boldsymbol{i}_s^{{(\alpha \beta )}}[k]+ \dfrac{T_s}{2}\boldsymbol{r}_1(1)\right\} + ( a_{21} + a_{22}\omega _r\boldsymbol{J} )\left\{ \boldsymbol{\psi }_s^{{(\alpha \beta )}}[k]+ \dfrac{T_s}{2}\boldsymbol{r}_1(2)\right\} + b_1 \boldsymbol{v}_s^{{(\alpha \beta )}}[k]\\ - R_s \left\{ \boldsymbol{i}_s^{{(\alpha \beta )}}[k]+ \dfrac{T_s}{2}\boldsymbol{r}_1(1)\right\} + \boldsymbol{v}_s^{{(\alpha \beta )}}[k]\end{bmatrix} , \\ \boldsymbol{r}_3 =&\begin{bmatrix} \left( a_{11} + a_{12}\omega _r\boldsymbol{J}\right) \left\{ \boldsymbol{i}_s^{{(\alpha \beta )}}[k]+ \dfrac{T_s}{2}\boldsymbol{r}_2(1)\right\} + ( a_{21} + a_{22}\omega _r\boldsymbol{J} )\left\{ \boldsymbol{\psi }_s^{{(\alpha \beta )}}[k]+ \dfrac{T_s}{2}\boldsymbol{r}_2(2)\right\} + b_1 \boldsymbol{v}_s^{{(\alpha \beta )}}[k]\\ - R_s \left\{ \boldsymbol{i}_s^{{(\alpha \beta )}}[k]+ \dfrac{T_s}{2}\boldsymbol{r}_2(1)\right\} + \boldsymbol{v}_s^{{(\alpha \beta )}}[k]\end{bmatrix} , \\ \boldsymbol{r}_4 =&\begin{bmatrix} \left( a_{11} + a_{12}\omega _r\boldsymbol{J}\right) \left\{ \boldsymbol{i}_s^{{(\alpha \beta )}}[k]+ {T_s}\boldsymbol{r}_3(1)\right\} + ( a_{21} + a_{22}\omega _r\boldsymbol{J} )\left\{ \boldsymbol{\psi }_s^{{(\alpha \beta )}}[k]+ {T_s}\boldsymbol{r}_3(2)\right\} + \boldsymbol{b}_1 v_s^{{(\alpha \beta )}}[k]\\ - R_s \left\{ \boldsymbol{i}_s^{{(\alpha \beta )}}[k]+ {T_s}2\boldsymbol{r}_3(1)\right\} + \boldsymbol{v}_s^{{(\alpha \beta )}}[k]\end{bmatrix} .\\ \end{aligned} \end{aligned}$$

## Predictive torque and flux control

The classical approach of model predictive control (MPC) in IM drives is the predictive torque and flux control (PTC). This technique uses the inherent discrete nature of the power converter to solve the optimization problem using a single cost function; therefore, it is classified within the finite control set MPC methods. Thus, the input is restricted to a finite set of discrete values. The discrete-time system model is evaluated for every possible actuation sequence and then compared with the reference in order to select the voltage vector that minimizes the cost function ^26,27^.

The standard PTC approach uses a single cost function built by a linear combination of the objective functions to determine the best voltage vector to select in the next sampling time. The torque and the stator flux linkage errors are included in one cost function through the use of weighting factors. Typically, the cost function is defined as

26 $$\begin{aligned} g = \left( \mathscr {T}_e^{(\text {ref})} - \mathscr {T}_e[k\!+\!1]\right) ^2 + \gamma \,\left( \Vert \boldsymbol{\psi }_s^{{(\alpha \beta )}}\Vert ^{(\text {ref})} - \Vert \boldsymbol{\psi }_s^{{(\alpha \beta )}}[k\!+\!1]\Vert \right) ^2 , \end{aligned}$$

where the superscript $^{(\text {ref})}$ denotes the reference value and $\Vert \cdot \Vert$ the euclidean norm of a vector, the weighting factor $\gamma$ is usually defined in function of the nominal values of torque and stator flux linkage $\gamma = \mathscr {T}_{\text {nom}}\,/\,{\psi }_{s,\text {nom}}^{{(\alpha \beta )}}$ ^26,28^. The cost function is evaluated for each voltage vector; for a 2-level voltage source converter (2L-VSC) there are seven non-redundant possible voltage vectors $\boldsymbol{v}_s^{(\alpha \beta )}\in \left\{ v_0,\dots ,v_6\right\}$.

The voltage that minimizes the cost function $\boldsymbol{v}_{\text {opt}}^{{(\alpha \beta )}}$ is applied in the next sampling interval

27 $$\begin{aligned} \boldsymbol{v}_{\text {opt}}^{{(\alpha \beta )}} = \arg \ \underset{\left\{ v_0,\dots ,v_6\right\} }{\min } g(\boldsymbol{v}_s^{{(\alpha \beta )}}) . \end{aligned}$$

**Fig. 1:**
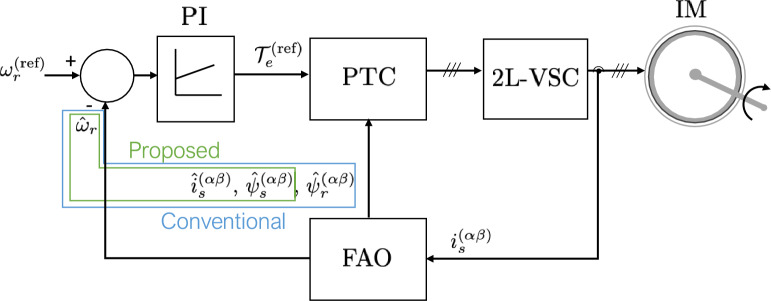
Proposed and conventional FAO–PTC scheme.

Finally, an external speed control loop is added to generate the torque reference for the PTC scheme. This loop is implemented using a PI controller, as shown in Fig. 1. The figure illustrates both the conventional and the proposed sensorless FAO–PTC control schemes. In the proposed approach, the stator current and stator flux linkage are estimated by the FAO, whereas the conventional scheme also requires estimating the rotor flux linkage, as its IM model is defined by these three variables ^28^. Consequently, the conventional method involves a more complex estimation and prediction process due to the inclusion of the rotor flux linkage among the state variables.

### Discrete-time models of prediction

Torque and stator flux linkage predictions, rely on the IM model, the accuracy of the prediction depends on the discretization of equation (3).

In conventional PTC, the IM dynamic is described using the stator current, rotor flux linkage, and stator flux linkage ^28^. As mentioned earlier, since currents and flux linkages are linearly dependent, the IM dynamic can be fully represented using only the stator current and stator flux linkage, reducing both the complexity of the model and the size of the state vector.

Unlike FAO discretization, in the proposed PTC, the mechanical dynamic is taken into account; therefore, the system to be discretized is non-linear. It is not necessary to predict the rotor shaft speed; however, the stator currents and stator flux linkages are predicted by considering the mechanical dynamics during the discretization.

In this section, stator currents and flux linkages discrete-time predictions for PTC are described

#### Euler

Euler-based PTC model is the same as FAO model (22).

#### Taylor

Taylor-based predictions are

28 $$\begin{aligned} \begin{aligned} \boldsymbol{i}_s^{{(\alpha \beta )}}[k\!+\!1]=&\left\{ T_s\,c_1 - \dfrac{T_s^2}{2}\left[ R_s\,a_{21} - a_{11}\,c_1 - \omega _r[k](a_{12}c_1 - R_s a_{22})\boldsymbol{J} - \dfrac{a_{12}}{H}\boldsymbol{J}(\mathscr {T}_e-\mathscr {T}_l)\right] + 1\right\} \boldsymbol{i}_s^{(\alpha \beta )}[k]\\&+ \left\{ T_s c_2 + \dfrac{T_s^2}{2} \left( c_1 c_2 + \dfrac{a_{22}}{H}(\mathscr {T}_e-\mathscr {T}_l)\boldsymbol{J}\right) \right\} \boldsymbol{\psi }_s^{ab}[k]\\&+ \left\{ T_s b_1 + \dfrac{T_s^2}{2}\left[ a_{21} + a_{11} b_1 + \omega _r[k](a_{22} + a_{12} b_1 )\boldsymbol{J}\right] \right\} \boldsymbol{v}_s^{{(\alpha \beta )}}[k]+ b_1\dfrac{T_s^2}{2}\frac{d}{dt}\boldsymbol{v}_s^{{(\alpha \beta )}} , \\ \boldsymbol{\psi }_s^{{(\alpha \beta )}}[k\!+\!1]=&\left\{ -R_s\left( T_s + \dfrac{T_s^2}{2}c_1 \right) \right\} \boldsymbol{i}_s^{(\alpha \beta )}[k]+ \left\{ 1 - \dfrac{T_s^2}{2}R_s c_2\right\} \boldsymbol{\psi }_s^{(\alpha \beta )}[k]+ \left\{ T_s - \dfrac{T_s^2}{2}R_s b_1\right\} \boldsymbol{v}_s^{{(\alpha \beta )}}[k]+ \dfrac{T_s^2}{2}\dfrac{d}{dt}\boldsymbol{v}_s^{{(\alpha \beta )}}. \end{aligned} \end{aligned}$$

Note that, $c_1$, $c_2$, $\mathscr {T}_e$ and $\mathscr {T}_l$ depend on instant $[k]$, i.e., $c_1[k]$, $c_2[k]$, $\mathscr {T}_e[k]$ and $\mathscr {T}_l[k]$.

#### RK 2

To predict stator current and stator flux linkage, speed estimation points are included in RK 2-based PTC

29 $$\begin{aligned} \nonumber \boldsymbol{r}_1 =&\begin{bmatrix} \left( a_{11} + ja_{12}\omega _r[k]\boldsymbol{J}\right) \boldsymbol{i}_s^{{(\alpha \beta )}}[k]+ ( a_{21} + a_{22}\omega _r[k]\boldsymbol{J} )\boldsymbol{\psi }_s^{{(\alpha \beta )}}[k]+ b_1 \boldsymbol{v}_s^{{(\alpha \beta )}}[k]\\ - R_s \boldsymbol{i}_s^{{(\alpha \beta )}}[k]+ \boldsymbol{v}_s^{{(\alpha \beta )}}[k]\\ \frac{1}{H}(\mathscr {T}_e[k]-\mathscr {T}_l[k]) \end{bmatrix} , \\ \boldsymbol{r}_2 =&\begin{bmatrix} \left( a_{11} + a_{12}\left\{ \omega _r[k]+ T_s\boldsymbol{r}_1(3)\right\} \boldsymbol{J}\right) \left\{ \boldsymbol{i}_s^{{(\alpha \beta )}}[k]+ T_s\boldsymbol{r}_1(1)\right\} + ( a_{21} + a_{22}\left\{ \omega _r[k]+ T_s\boldsymbol{r}_1(3)\right\} \boldsymbol{J})\left\{ \boldsymbol{\psi }_s^{{(\alpha \beta )}}[k]+ T_s\boldsymbol{r}_1(2)\right\} + b_1 \boldsymbol{v}_s^{{(\alpha \beta )}}[k]\\ - R_s \left\{ \boldsymbol{i}_s^{{(\alpha \beta )}}[k]+ T_s\boldsymbol{r}_1(1)\right\} + \boldsymbol{v}_s^{{(\alpha \beta )}}[k]\\ \frac{1}{H}\left( 1.5p\left( \left\{ \boldsymbol{\psi }_s^{{(\alpha \beta )}}[k]+ T_s\boldsymbol{r}_1(2)\right\} \times \left\{ \boldsymbol{i}_s^{{(\alpha \beta )}}[k]+ T_s\boldsymbol{r}_1(1)\right\} \right) - \mathscr {T}_l[k]\right) \end{bmatrix}. \end{aligned}$$

#### RK 4

Similarly to RK 2-based predictions, speed estimation points are also included in RK 4-based PTC. RK 4 estimation point $\boldsymbol{r}_1$ is the same as RK 2, the others are

30 $$\begin{aligned} \nonumber \boldsymbol{r}_2 =&\begin{bmatrix} \left( a_{11} + a_{12}\left\{ \omega _r[k]+ \dfrac{T_s}{2}\boldsymbol{r}_1(3)\right\} \boldsymbol{J}\right) \left\{ \boldsymbol{i}_s^{{(\alpha \beta )}}[k]+ \dfrac{T_s}{2}\boldsymbol{r}_1(1)\right\} + ( a_{21} + a_{22}\left\{ \omega _r[k]+ \dfrac{T_s}{2}\boldsymbol{r}_1(3)\right\} \boldsymbol{J})\left\{ \boldsymbol{\psi }_s^{{(\alpha \beta )}}[k]+ \dfrac{T_s}{2}\boldsymbol{r}_1(2)\right\} + b_1 \boldsymbol{v}_s^{{(\alpha \beta )}}[k]\\ - R_s \left\{ \boldsymbol{i}_s^{{(\alpha \beta )}}[k]+ \dfrac{T_s}{2}\boldsymbol{r}_1(1)\right\} + \boldsymbol{v}_s^{{(\alpha \beta )}}[k]\\ \frac{1}{H}\left( 1.5p\left( \left\{ \boldsymbol{\psi }_s^{{(\alpha \beta )}}[k]+ \dfrac{T_s}{2}\boldsymbol{r}_1(2)\right\} \times \left\{ \boldsymbol{i}_s^{{(\alpha \beta )}}[k]+ \dfrac{T_s}{2}\boldsymbol{r}_1(1)\right\} \right) - \mathscr {T}_l[k]\right) \end{bmatrix} , \\ \nonumber \boldsymbol{r}_3 =&\begin{bmatrix} \left( a_{11} + a_{12}\left\{ \omega _r[k]+ \dfrac{T_s}{2}\boldsymbol{r}_2(3)\right\} \boldsymbol{J}\right) \left\{ \boldsymbol{i}_s^{{(\alpha \beta )}}[k]+ \dfrac{T_s}{2}\boldsymbol{r}_2(1)\right\} + ( a_{21} + a_{22}\left\{ \omega _r[k]+ \dfrac{T_s}{2}\boldsymbol{r}_2(3)\right\} \boldsymbol{J})\left\{ \boldsymbol{\psi }_s^{{(\alpha \beta )}}[k]+ \dfrac{T_s}{2}\boldsymbol{r}_2(2)\right\} + b_1 \boldsymbol{v}_s^{{(\alpha \beta )}}[k]\\ - R_s \left\{ \boldsymbol{i}_s^{{(\alpha \beta )}}[k]+ \dfrac{T_s}{2}\boldsymbol{r}_2(1)\right\} + \boldsymbol{v}_s^{{(\alpha \beta )}}[k]\\ \frac{1}{H}\left( 1.5p\left( \left\{ \boldsymbol{\psi }_s^{{(\alpha \beta )}}[k]+ \dfrac{T_s}{2}\boldsymbol{r}_2(2)\right\} \times \left\{ \boldsymbol{i}_s^{{(\alpha \beta )}}[k]+ \dfrac{T_s}{2}\boldsymbol{r}_2(1)\right\} \right) - \mathscr {T}_l[k]\right) \end{bmatrix} , \\ \boldsymbol{r}_4 =&\begin{bmatrix} \left( a_{11} + a_{12}\left\{ \omega _r[k]+ {T_s}\boldsymbol{r}_3(3)\right\} \boldsymbol{J}\right) \left\{ \boldsymbol{i}_s^{{(\alpha \beta )}}[k]+ {T_s}\boldsymbol{r}_3(1)\right\} + ( a_{21} + a_{22}\left\{ \omega _r[k]+ {T_s}\boldsymbol{r}_3(3)\right\} \boldsymbol{J})\left\{ \boldsymbol{\psi }_s^{{(\alpha \beta )}}[k]+ {T_s}\boldsymbol{r}_3(2)\right\} + b_1 \boldsymbol{v}_s^{{(\alpha \beta )}}[k]\\ - R_s \left\{ \boldsymbol{i}_s^{{(\alpha \beta )}}[k]+ {T_s}\boldsymbol{r}_2(1)\right\} + \boldsymbol{v}_s^{{(\alpha \beta )}}[k]\\ \frac{1}{H}\left( 1.5p\left( \left\{ \boldsymbol{\psi }_s^{{(\alpha \beta )}}[k]+ T_s\boldsymbol{r}_3(2)\right\} \times \left\{ \boldsymbol{i}_s^{{(\alpha \beta )}}[k]+ T_s\boldsymbol{r}_3(1)\right\} \right) - \mathscr {T}_l[k]\right) \end{bmatrix} . \end{aligned}$$

## Hardware-in-the-loop results

The different discretization methods were incorporated into both the FAO and PTC schemes. The Hardware-in-the-Loop (HIL) results were obtained using the PLECS RT-Box 3. For the HIL simulations, the base discretization time was set to $T_{\text {base}} = 4\,\mu \text {s}$, while the discretization time used in the FAO and MPC algorithms was $T_s = 40\,\mu \text {s}$. A 4 kW IM was employed, with nominal parameters: $V = 380$ V, $f = 50$ Hz, $\omega _r = 151.84$ rad/s (1450 rpm), $R_s = R_r = 1.1~\Omega$, $L_s = L_r = 164$ mH, $L_m = 160$ mH, and $H = 0.08$ kg$\cdot$m$^2$.

**Fig. 2:**
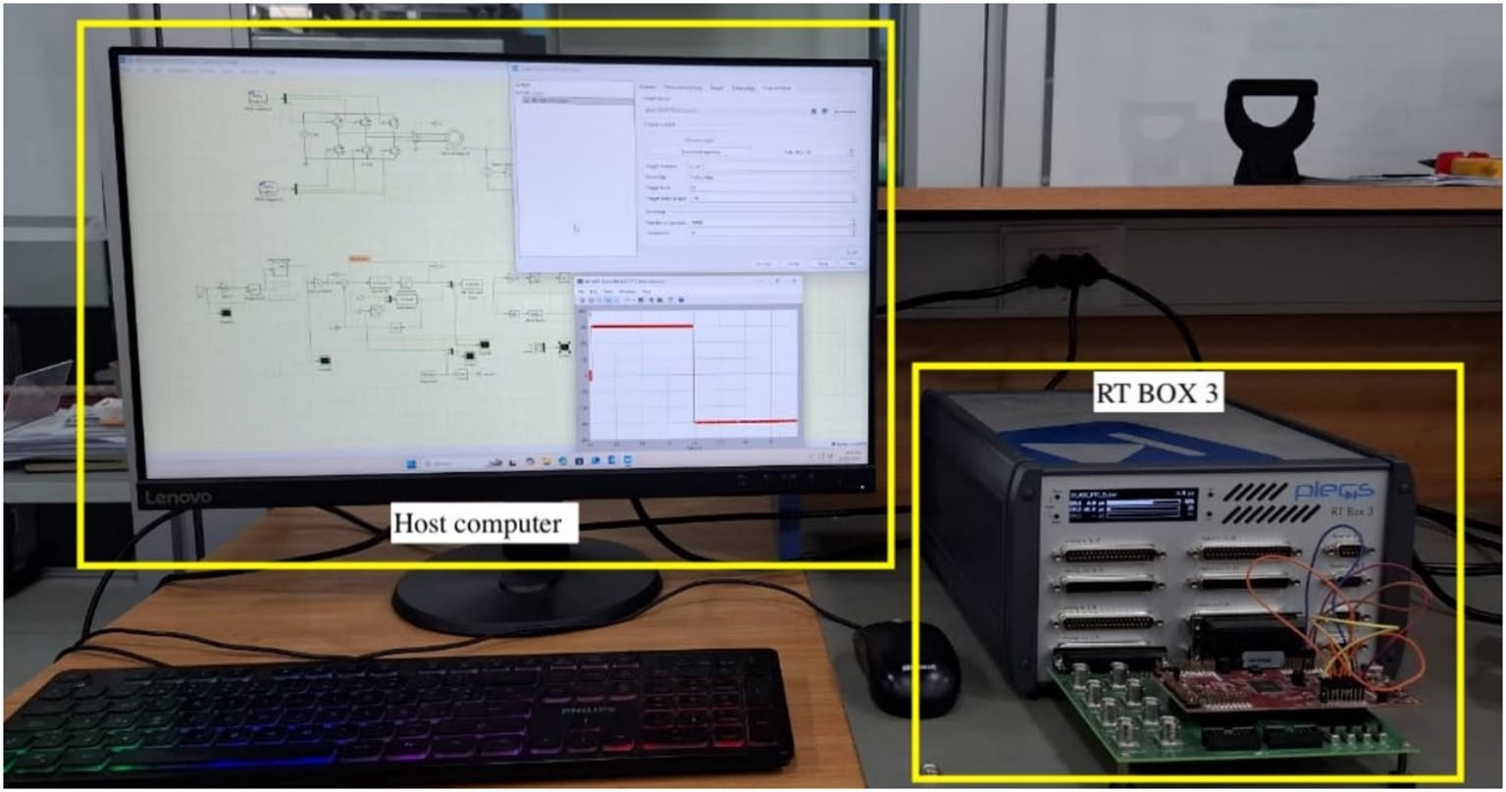
HIL setup, showing the PLECS RT-Box 3 and the host computer used for real-time simulations.

Figure 2 shows the complete HIL setup adopted for validation, which comprises the PLECS RT-Box 3 and a host computer used to run the simulations, monitoring, and data acquisition. This HIL platform enables the real-time emulation of the physical plant and the control system, allowing the proposed schemes to be evaluated under conditions that closely resemble those of a real system. The data obtained from the HIL experiments were subsequently used for post-processing, figure generation, and performance analysis.

Within the HIL framework, the induction machine and power converter were implemented on one real-time CPU core, representing the physical plant, while a separate CPU core was dedicated exclusively to the execution of the sensorless control scheme, representing the digital control system. In this configuration, the execution time of the physical plant model is one order of magnitude smaller than that of the control algorithm, distinguishing the continuous-time dynamics of the physical plant from the discrete-time implementation of the digital controller. The control signals generated by the sensorless controller were interfaced with the power converter gating signals through the digital I/O channels of the RT-Box, enabling an accurate real-time emulation of the interaction between the control and power stages.

To compare the discretization methods in the FAO, a direct start from a 50 Hz, 380 V grid is first performed. Then, the proposed sensorless FAO–PTC scheme is evaluated using different PTC models under a 540 V dc-link supply, applying two step changes that drive the machine from standstill to nominal speed and then reverse the rotation, reaching nominal speed again. In both cases, the load torque is modelled as a viscous friction torque linearly proportional to the rotor shaft speed, $\mathscr {T}_l = K_{\mathscr {T}}\,\omega _r$, such that at nominal speed it develops the nominal torque.

Additionally, to ensure a fair comparison among the different FAO models, the FAO performance was evaluated under different PI gain settings. The baseline gains were taken from ^11^, originally designed for the conventional Euler-based FAO model ($K_p$ = 1.8, $K_i$ = 1200). To assess the influence of the PI gains on the adaptive speed scheme, two additional gain sets were obtained by proportionally scaling both $K_p$ and $K_i$ while preserving a constant gain ratio. This scaling strategy ensures that the discrete-time controller zero remains invariant. From a control perspective, proportional gain scaling shifts the closed-loop poles while maintaining the relative damping characteristics of the system, enabling a systematic evaluation of FAO performance across different bandwidth levels without altering the structural stability or damping profile of the adaptive law. Consequently, any observed performance variations can be attributed to the observer model dynamics rather than to gain-selection biases.

Moreover, since the IM model is inherently stable and to avoid introducing additional tuning parameters that could bias the comparison, the observer gain matrix is set to zero by selecting $\eta = 1$, which corresponds to the Solution II gain set.

Finally, the execution times of the different FAO–PTC schemes were obtained from their real-time implementation on the RT-Box 3 (1.5 GHz CPU). The processor clock counter was read immediately before and after each FAO and PTC execution within a sampling interval, and the difference in clock ticks was converted into execution time using the CPU frequency.

### FAO results

The machine is directly connected to the grid and different-modelled FAO estimates the stator currents, stator flux linkages and speed. To evaluate the performance of the estimations, a root mean squared error between the estimation $\boldsymbol{\hat{x}}$ and the actual value $\boldsymbol{x}$ is computed

31 $$\begin{aligned} \text {RMSE}\{\boldsymbol{x}\} = \sqrt{\frac{1}{N}\sum _{k=1}^N\left( \boldsymbol{x}[k]- \boldsymbol{\hat{x}}[k]\right) ^2} , \end{aligned}$$

where *N* is the total number of samples.

The proposed FAO discrete-time models were compared with the conventional FAO, which is based on stator current and rotor flux linkage. In the conventional approach, the state is expanded to also estimate the stator flux linkage.

**Fig. 3:**
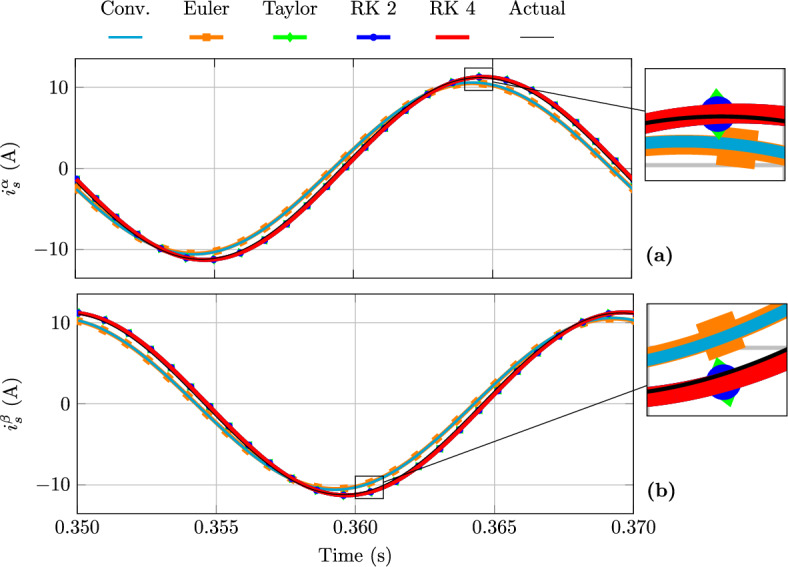
Stator current FAO estimation at steady state from a direct start: (**a**) $\alpha$-axis and (**b**) $\beta$-axis components.

**Fig. 4:**
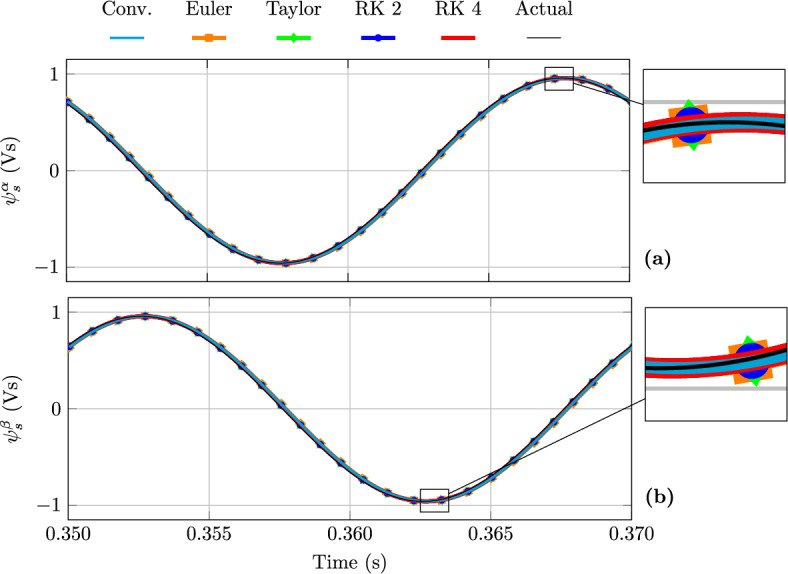
Stator flux linkage FAO estimation at steady state from a direct start: (**a**) $\alpha$-axis and (**b**) $\beta$-axis components.

Figure 3 illustrates the steady-state behaviour of the stator current, while Fig. 4 shows the flux linkage. In Fig. 3 an appreciable phase and magnitude error in the stator currents estimation is observed for both the conventional and Euler-based FAO model; conversely, the Taylor and RK FAO models exhibit negligible phase and magnitude errors in the stator current estimation. On the other hand, Fig. 4 shows that the stator flux linkage estimation errors are negligible for all the considered models.

**Fig. 5:**
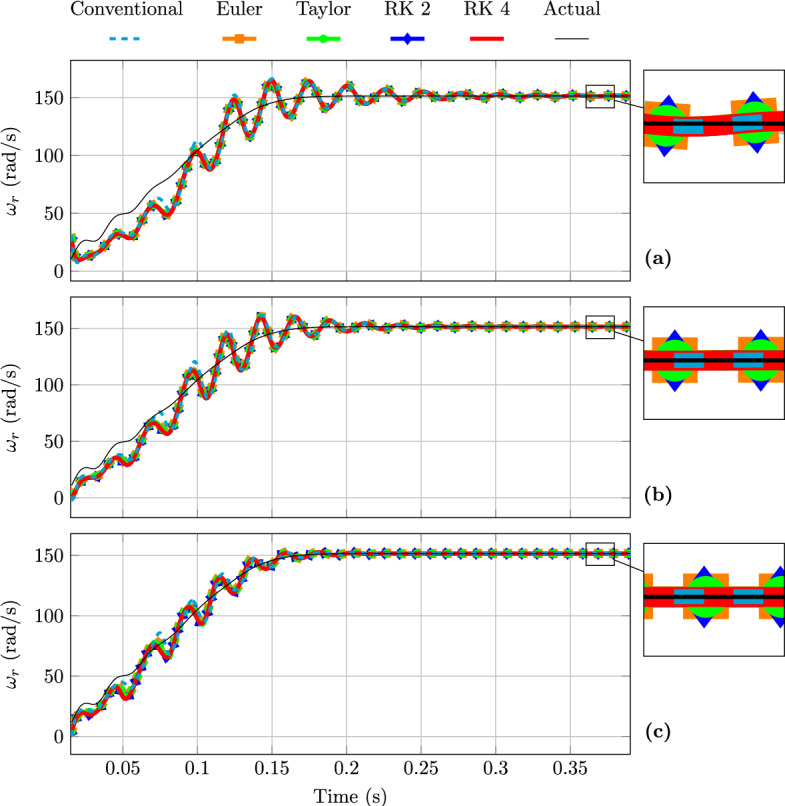
Rotor shaft speed FAO estimation from a direct start for different PI gains. (**a**) $K_p = 0.9$, $K_i = 600$. (**b**) $K_p = 1.8$, $K_i = 1200$. (**c**) $K_p = 3.6$, $K_i = 2400$.

Figure 5 illustrates the FAO rotor shaft speed estimation during a direct start for three different PI gain configurations. Increasing the PI gains results in a faster convergence of the FAO towards the steady state, whereas reducing the gains leads to a slower convergence accompanied by increased oscillatory behaviour during the transient, which in turn increases the settling time. For a given PI gain set, all FAO models exhibit a similar dynamic response, indicating that the transient FAO response behaviour is primarily governed by the PI gains rather than the discrete-time model used.

Tables 1, 2, and 3 summarize the RMSE obtained for each FAO model using the same PI gain configurations considered in Fig. 5, separately reporting the transient, steady-state, and overall responses. During the transient stage, for a given PI gain set, all models exhibit comparable RMSE values, indicating similar estimation accuracy during the startup phase. As the PI gains are increased, the transient RMSE decreases for all models; however, at higher PI gain settings, small differences emerge in the RMSE of the stator flux linkage and rotor shaft speed between the conventional and proposed models.

In steady state, the RMSE of the proposed second- and higher-order models varies significantly with the PI gains, whereas the conventional and Euler-based models exhibit RMSE values that are largely insensitive to the tested PI gain settings. In particular, the proposed Euler-based FAO yields a steady-state speed RMSE approximately three times higher than that of the conventional model, while simultaneously reducing the stator flux linkage estimation error by about 50%. Furthermore, the steady-state RMSE of the RK 2 and RK 4 models does not decrease proportionally with increasing PI gains. Nevertheless, the speed estimation error is reduced by up to 96% with the Taylor-based FAO and by up to 86% with the Runge–Kutta-based models relative to the conventional approach.

Finally, when considering the overall response, including both transient and steady-state operation, all models exhibit similar RMSE levels for a given PI gain setting, with the RMSE decreasing as the PI gains are increased; nevertheless, the conventional model consistently shows slightly higher RMSE values compared to the proposed models.

**Table 1:** RMSE of different FAO models using $K_p = 0.9$, $K_i = 600$.

(a) Transient			
FAO Model	$\Vert i_s^{\alpha \beta } \Vert$	$\Vert \psi _s^{\alpha \beta }\Vert$ ($\times 10^{-3}$)	$\omega _r$
Conv.	4.674	77.658	21.872
Euler	4.623	73.395	21.277
Taylor	4.537	73.448	21.285
RK 2	4.561	73.490	21.301
RK 4	4.561	73.489	21.300

(b) Steady–state			
FAO Model	$\Vert i_s^{\alpha \beta } \Vert$ ($\times 10^{-3}$)	$\Vert \psi _s^{\alpha \beta }\Vert$ ($\times 10^{-6}$)	$\omega _r$ ($\times 10^{-3}$)
Conv.	689.023	483.605	32.729
Euler	757.434	233.708	76.244
Taylor	18.376	151.657	14.650
RK 2	5.018	53.972	10.378
RK 4	5.820	54.532	9.626

(c) Total			
FAO Model	$\Vert i_s^{\alpha \beta } \Vert$	$\Vert \psi _s^{\alpha \beta }\Vert$ ($\times 10^{-3}$)	$\omega _r$
Conv.	3.315	54.458	15.338
Euler	3.287	51.468	14.920
Taylor	3.182	51.505	14.926
RK 2	3.198	51.535	14.937
RK 4	3.199	51.533	14.937

**Table 2:** RMSE of different FAO models using $K_p = 1.8$, $K_i = 1200$.

(a) Transient			
FAO Model	$\Vert i_s^{\alpha \beta } \Vert$	$\Vert \psi _s^{\alpha \beta }\Vert$ ($\times 10^{-3}$)	$\omega _r$
Conv.	2.626	51.559	19.300
Euler	2.497	48.054	18.526
Taylor	2.393	48.434	18.544
RK 2	2.439	48.699	18.570
RK 4	2.439	48.696	18.570

(b) Steady–state			
FAO Model	$\Vert i_s^{\alpha \beta } \Vert$ ($\times 10^{-3}$)	$\Vert \psi _s^{\alpha \beta }\Vert$ ($\times 10^{-6}$)	$\omega _r$ ($\times 10^{-3}$)
Conv.	689.387	474.799	31.144
Euler	775.791	156.931	87.140
Taylor	11.052	115.682	5.595
RK 2	18.132	47.572	14.005
RK 4	17.982	53.063	10.785

(c) Total			
FAO Model	$\Vert i_s^{\alpha \beta } \Vert$	$\Vert \psi _s^{\alpha \beta }\Vert$ ($\times 10^{-3}$)	$\omega _r$
Conv.	1.906	36.157	13.534
Euler	1.836	33.698	12.992
Taylor	1.678	33.964	13.004
RK 2	1.710	34.150	13.022
RK 4	1.710	34.148	13.022

**Table 3:** RMSE of different FAO models using $K_p = 3.6$, $K_i = 2400$.

(a) Transient			
FAO Model	$\Vert i_s^{\alpha \beta } \Vert$	$\Vert \psi _s^{\alpha \beta }\Vert$ ($\times 10^{-3}$)	$\omega _r$
Conv.	1.349	28.172	16.564
Euler	1.219	24.556	15.706
Taylor	1.009	25.179	15.732
RK 2	1.069	25.764	15.784
RK 4	1.069	25.759	15.784

(b) Steady–state			
FAO Model	$\Vert i_s^{\alpha \beta } \Vert$ ($\times 10^{-3}$)	$\Vert \psi _s^{\alpha \beta }\Vert$ ($\times 10^{-6}$)	$\omega _r$ ($\times 10^{-3}$)
Conv.	689.419	654.133	31.141
Euler	788.900	109.115	95.626
Taylor	5.472	93.406	1.637
RK 2	28.008	86.664	21.010
RK 4	27.140	89.282	17.303

(c) Total			
FAO Model	$\Vert i_s^{\alpha \beta } \Vert$ ($\times 10^{-3}$)	$\Vert \psi _s^{\alpha \beta }\Vert$ ($\times 10^{-3}$)	$\omega _r$
Conv.	1066.012	19.761	11.615
Euler	1023.145	17.220	11.014
Taylor	707.589	17.657	11.032
RK 2	750.054	18.067	11.069
RK 4	750.021	18.064	11.068

The execution time per time step of the different FAO models is summarized in Table 4. The proposed Euler-based FAO significantly reduces the execution time compared to the conventional observer, resulting in an approximate 38% reduction in computational cost. In contrast, the Taylor-based FAO exhibits an execution time about 18% higher than that of the conventional approach, while delivering the best steady-state performance among the evaluated methods. Finally, the RK 4-based FAO requires more than twice the execution time of the conventional model.

**Table 4:** Average execution time per time step of different FAO models.

FAO Model	Execution time
Conventional	0.161 $\mu$s
Euler	0.099 $\mu$s
Taylor	0.191 $\mu$s
RK 2	0.202 $\mu$s
RK 4	0.334 $\mu$s

### PTC results

In the PTC control scheme, the controlled variables are fed back through the FAO estimation. Additionally, an external speed control loop is added to provide the torque reference for the PTC scheme. The speed control loop is implemented using a PI controller, systematically designed based on the mechanical transfer function 1/*Hs*, with $1/\sqrt{2}$ as damping factor and 200 Hz as bandwidth. Additionally, to control stator flux linkage, a fixed value of $\Vert \boldsymbol{\psi }_s^{{(\alpha \beta )}}\Vert ^{(\text {ref})} = 0.67$ Vs was selected throughout the simulations.

To evaluate the performance of the proposed sensorless FAO–PTC scheme, a sudden speed reference step is applied to the nominal operating speed, followed by a direction reversal at 2 seconds. The RMSE,

32 $$\begin{aligned} \text {RMSE}\{\boldsymbol{x}\} = \sqrt{\frac{1}{N}\sum _{k=1}^N\left( \boldsymbol{x}^{(\text {ref})}[k]- \boldsymbol{x}[k]\right) ^2} , \end{aligned}$$

allows for quantifying torque ripple and assessing the deviation of the actual flux linkage and rotor shaft speed from their reference values.

#### Conventional FAO–PTC comparison

The conventional FAO estimates the stator current and rotor flux linkage ^11^. Since the PTC strategy directly controls the stator flux linkage, the state vector can be expanded to estimate these flux linkages using the FAO without altering the observer structure. In the conventional PTC, the stator current is predicted based on the rotor flux linkage. In contrast, the proposed approach reformulates the IM model within both the FAO and PTC schemes, thereby eliminating the need to estimate the rotor flux linkage. For a fair comparison, both the conventional and proposed models employ the Euler discretization method in the FAO and PTC implementations.

Table 5 summarizes the steady-state performance, including the torque ripple and the deviations of speed and stator flux linkage magnitude from their reference values. As shown in the table, both the conventional and proposed methods achieve comparable performance under steady-state conditions.

**Fig. 6:**
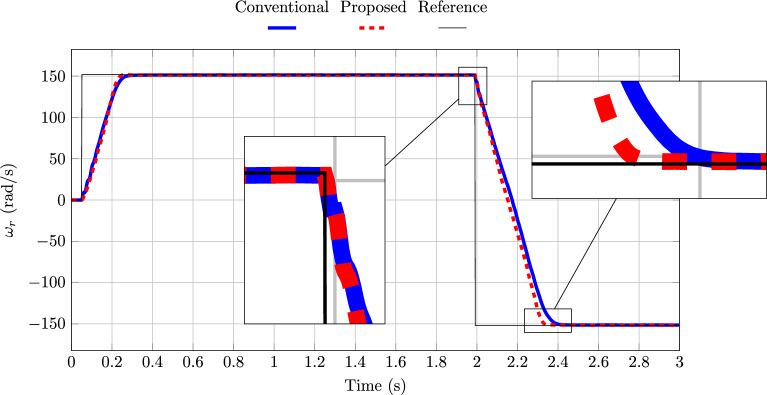
Rotor shaft speed for the conventional and the proposed Euler-based FAO–PTC.

As shown in Fig. 6, the proposed FAO–PTC model reaches steady state approximately 0.06 s earlier, corresponding to about 1500 sampling intervals. This is consistent with the lower transient RMSE observed for the proposed Euler-based FAO in Table 2 and highlights its faster transient response compared to the conventional scheme. In terms of ripple magnitude and reference-tracking accuracy, both methods exhibit similar steady-state behaviour. However, the proposed FAO–PTC scheme not only achieves quicker convergence to the speed reference but also reduces the number of estimated variables by two, thereby simplifying the overall implementation.

**Table 5:** Steady-state RMSE of the conventional and proposed FAO–PTC schemes, fully discretized using Euler.

Model	$\mathscr {T}_e$	$\omega _r$	$\Vert \boldsymbol{\psi }_s^{{(\alpha \beta )}} \Vert$
Conventional	2.2979	0.5361	0.1637
Proposed	2.3436	0.5545	0.1552

Table 6 reports the execution times of the conventional and proposed sensorless FAO–PTC schemes, both discretized using the Euler method. As observed, the proposed FAO–PTC scheme exhibits an approximately 16% higher execution time than the conventional one. This increase occurs despite the fact that, as shown in Table 4, the proposed FAO alone requires a lower execution time than the conventional FAO, since the latter estimates stator current, stator flux linkage, and rotor flux linkage. The higher execution time of the proposed FAO–PTC scheme is therefore mainly attributed to the PTC stage, where the proposed formulation involves a slightly larger number of mathematical operations for stator current prediction compared to the conventional scheme.

**Table 6:** Average execution time per time step of the conventional and proposed sensorless FAO–PTC schemes, fully discretized using Euler.

Model	Execution time
Conventional	0.590 $\mu$s
Proposed	0.684 $\mu$s

#### Proposed FAO–PTC Comparison

The different discrete-time models of the PTC scheme were compared, while the Taylor-based model was employed for the FAO in all cases. Table 7 presents the torque ripple, along with the deviations of speed and stator flux linkage magnitude from their reference values at steady state. Specifically, Table 5 reports the results of the proposed FAO–PTC scheme when both the FAO and the PTC are fully discretized using the Euler method, whereas Table 7 reports the results for the proposed FAO–PTC scheme with a Taylor-discretized FAO and an Euler-discretized PTC. Under these conditions, employing a more accurate observer model in the proposed Euler-based PTC scheme results in approximately a 10% reduction in torque ripple and in the RMSE of the stator flux linkage magnitude, as well as a 40% decrease in the steady-state speed error.

Furthermore, Table 7 indicates that the Taylor-based model achieves the best steady-state performance in terms of speed error and stator flux linkage magnitude deviation, which are approximately 10% and 14% lower, respectively, than those obtained with the Euler- and RK-based PTC models; however, this improvement comes at the expense of an approximately 5% increase in torque ripple compared to the Euler-based approach. This increased torque ripple can be attributed to the explicit inclusion of the stator voltage derivative in the Taylor-based prediction model. By using the 2L-VSC, the stator voltage exhibits abrupt transitions between 0 and $v_{dc}$, making its time derivative highly sensitive. This sensitivity directly affects the current and flux linkage predictions, and its impact is further amplified in the electromechanical torque computation, which involves the cross product of the stator current and flux linkage vectors.

On the other hand, despite its higher order, the RK 4 method performs marginally worse than the second-order RK 2 model. This behaviour indicates that neglecting future time steps in the prediction process considerably limits the achievable accuracy of the RK-based approaches.

**Fig. 7:**
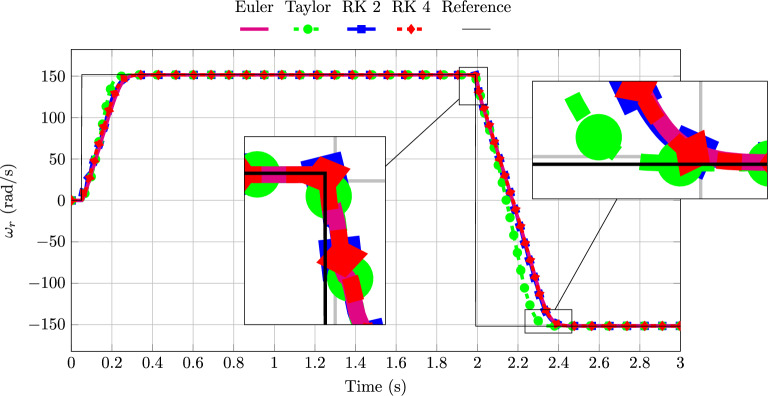
Rotor shaft speed for different discrete-time models of the proposed FAO–PTC.

Figure 7 shows the machine speed responses obtained using different IM discrete-time models within the PTC control scheme, together with the speed reference. The Taylor-based model exhibits the fastest transient response, reaching the reverse speed approximately 0.08 seconds earlier than the other methods, which corresponds to about 2000 sampling intervals. In contrast, the RK models display a dynamic performance similar to that of the Euler approach, as they do not account for future input values in the control prediction. Consequently, despite their higher numerical order, their behaviour more closely resembles that of the Euler model rather than the Taylor model.

**Table 7:** Steady-state RMSE of the proposed FAO–PTC scheme with a Taylor-discretized FAO, for different PTC models.

PTC Model	$\mathscr {T}_e$	$\omega _r$	$\Vert \boldsymbol{\psi }_s^{{(\alpha \beta )}} \Vert$
Euler	2.1277	0.3270	0.1385
Taylor	2.2520	0.2824	0.1246
RK 2	2.0506	0.3267	0.1355
RK 4	2.0774	0.3279	0.1368

The execution times of the proposed sensorless scheme using the Taylor-based FAO with different PTC models are reported in Table 8. As expected, the RK 4 implementation naturally exhibits the highest execution time due to its increased numerical complexity. In contrast, the second-order methods, RK 2 and Taylor, present very similar execution times; however, on average, the Taylor-based approach requires slightly more computational time than RK 2.

**Table 8:** Average execution time per time step of the proposed FAO–PTC scheme with a Taylor-discretized FAO, for different PTC models.

PTC Model	Execution time
Euler	0.771 $\mu$s
Taylor	1.040 $\mu$s
RK 2	0.959 $\mu$s
RK 4	1.608 $\mu$s

## Conclusions

The proposed reformulation of the FAO–PTC framework removes the requirement to estimate rotor flux linkage, which reduces the number of state variables and simplifies both the observer and controller structures. This leads to a more compact and coherent formulation that is well suited for real-time implementation in sensorless induction machine drives.

In the observer stage, the use of higher-order discrete-time models such as Taylor, RK 2 and RK 4 significantly improves the estimation accuracy of stator current, stator flux linkage and rotor shaft speed. The proposed Euler-based FAO further improves stator flux estimation relative to the conventional Euler formulation in steady state, although it increases the speed estimation error in steady-state. Among all evaluated discrete-time observer models, the Taylor-based FAO provides the best rotor shaft speed estimation overall. Although RK 4 is theoretically a fourth-order method, its performance remains close to that of second-order schemes such as Taylor and RK 2, since its discretization does not incorporate future input values, which limits its achievable accuracy in the considered control framework. In terms of execution time, the proposed Euler-based model achieves a lower average execution time than the conventional Euler formulation, while the Taylor-based FAO exhibits only a slightly higher computational cost, remaining one of the most efficient alternatives when jointly considering execution time and RMSE performance.

Focusing specifically on the FAO performance, and for a given PI tuning, all observer models exhibit very similar transient behaviour, indicating that the transient response is primarily governed by the PI gains rather than by the choice of discrete-time model. In steady state, however, differences among the models become more pronounced. In terms of speed estimation, the proposed Euler-based FAO exhibits poorer steady-state performance than the conventional formulation. The RK- and Taylor-based FAO models show steady-state behaviour that depends on the selected PI gains; among the RK-based approaches, RK 4 achieves an RMSE comparable to RK 2. Despite these differences, when both transient and steady-state performances are jointly considered, the conventional FAO exhibits a slightly inferior overall performance compared to the proposed models.

A direct comparison between the conventional and proposed FAO–PTC schemes, both discretized using Euler, shows that they achieve comparable steady-state performance in terms of torque ripple and reference tracking. However, the proposed scheme demonstrated a substantially faster transient response, reaching steady state quicker. In terms of execution time, it is worth noting that, despite eliminating the need to estimate rotor flux linkage, the proposed FAO–PTC scheme fully discretized using Euler requires a slightly higher average execution time than the conventional Euler-based FAO–PTC. This is mainly attributed to the increased number of mathematical operations introduced by the reformulated model, which, combined with the evaluation of all possible voltage vectors within each sampling interval inherent to the PTC strategy, leads to a modest increase in computational burden.

The influence of observer accuracy on PTC performance is also evident. When only the observer is improved, the PTC discretized by Euler already exhibits a marked enhancement in control performance. Additional improvements are obtained when the PTC is discretized using the Taylor model, which produces the fastest dynamic response among all the evaluated schemes. In contrast, the PTC discretized using RK 2 and RK 4 shows transient and steady-state behaviour similar to the Euler model, since these methods do not account for future input values in their discretization procedure, which limits their achievable accuracy.

The limited advantage of RK 4 over RK 2 can be explained by the assumptions underlying the derivation of the Runge–Kutta coefficients. The weighting coefficients associated with the intermediate solution estimates $\boldsymbol{r}$ are selected to match the local truncation error of second- and fourth-order Taylor series expansions under the assumption of sufficiently smooth system dynamics and continuously varying inputs. However, in the considered control framework, the zero-order hold assumption and the lack of knowledge of future input values violate these conditions. This absence of future input information introduces an additional approximation error that, when combined with the specific weighting of the intermediate stages in RK 4, prevents the method from achieving a tangible accuracy improvement despite its higher theoretical order.

When the estimation accuracy results are jointly analysed with the execution-time measurements, a clear trade-off emerges. Although RK 4 provides similar results in steady-state RMSE compared to RK 2, the former yields significantly higher computational burden. In contrast, the Taylor-based FAO offers a more favourable balance between estimation accuracy and execution time, achieving competitive or superior steady-state performance at a substantially lower computational cost. From a practical implementation perspective, the Taylor-based discretization therefore represents the most attractive alternative among the evaluated higher-order schemes.

Overall, the proposed reformulated FAO–PTC framework, particularly when combined with higher-order discrete-time models, provides an effective balance between estimation accuracy, dynamic performance and implementation simplicity. These characteristics make it a strong and practical candidate for real-time sensorless control of induction machine drives.

## Data Availability

The datasets generated during and/or analysed during the current study are available from the corresponding author on reasonable request.
